# Therapeutic Potential of Intrabodies for Cancer Immunotherapy: Current Status and Future Directions

**DOI:** 10.3390/antib11030049

**Published:** 2022-07-18

**Authors:** Thomas Böldicke

**Affiliations:** Department Structure and Function of Proteins, Helmholtz Centre for Infection Research, 38124 Braunschweig, Germany; thomas.boeldicke@helmholtz-hzi.de

**Keywords:** TAAs, neoantigens, intrabodies, cancer immunotherapy, nanoparticles, therapeutic mRNA, adeno-associated virus

## Abstract

Tumor cells are characterized by overexpressed tumor-associated antigens or mutated neoantigens, which are expressed on the cell surface or intracellularly. One strategy of cancer immunotherapy is to target cell-surface-expressed tumor-associated antigens (TAAs) with therapeutic antibodies. For targeting TAAs or neoantigens, adoptive T-cell therapies with activated autologous T cells from cancer patients transduced with novel recombinant TCRs or chimeric antigen receptors have been successfully applied. Many TAAs and most neoantigens are expressed in the cytoplasm or nucleus of tumor cells. As alternative to adoptive T-cell therapy, the mRNA of intracellular tumor antigens can be depleted by RNAi, the corresponding genes or proteins deleted by CRISPR-Cas or inactivated by kinase inhibitors or by intrabodies, respectively. Intrabodies are suitable to knockdown TAAs and neoantigens without off-target effects. RNA sequencing and proteome analysis of single tumor cells combined with computational methods is bringing forward the identification of new neoantigens for the selection of anti-cancer intrabodies, which can be easily performed using phage display antibody repertoires. For specifically delivering intrabodies into tumor cells, the usage of new capsid-modified adeno-associated viruses and lipid nanoparticles coupled with specific ligands to cell surface receptors can be used and might bring cancer intrabodies into the clinic.

## 1. Introduction

Cancer immunotherapy with monoclonal antibodies or antibody fragments comprises the targeting of antibodies to extracellular or intracellular tumor-associated antigens (TAAs) or mutated neoantigens [1,2,3]. In contrast to TAAs, neoantigens comprise tumor-specific mutations and are not expressed by normal cells. The binding of tumor-specific antibodies to extracellular TAAs or neoantigens activates natural killer cells, macrophages or the complement system, leading to the destruction of the tumor cells. In addition to naked monoclonal antibodies, bispecific antibodies, immunotoxins, immunocytokines and engineered CAR-T cells with a TAA or neoantigen-specific antibody fragment or TCR-like antibody fused to T-cell signal domains can be successfully applied [4,5].

In contrast to targeting antibodies to extracellular tumor antigens, it is now also possible to target intracellular oncogenic proteins. Two approaches exist which use antibodies: Antibodies that bind cell surface major histocompatibility complex class I (MHC-I)-presented peptides derived from intracellular proteins, T-cell receptor mimics (TCRm antibodies or TCR-like antibodies) [6] or intracellular antibodies that can be produced inside tumor cells and inhibit the function of oncogenic proteins [7,8].

**Figure 1:**
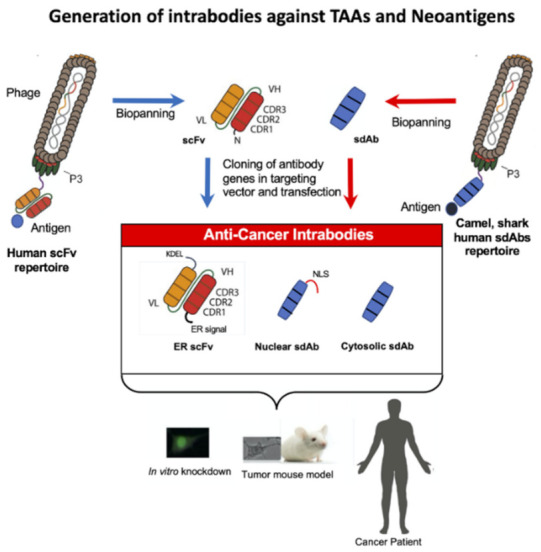
Schematic overview of phage display technology for the generation of intrabodies in cancer therapy.

Intracellular neoantigens and particularly neoepitopes, major histocompatibility complex (MHC)-bound peptides that arise from tumor-specific neoantigen mutations, are promising targets for adoptive T-cell therapy with autologous tumor-infiltrating lymphocytes expressing endogenous TCRs, gene-modified T cells expressing novel T-cell receptors or chimeric antigen receptor (CAR) T cells comprising recombinant antibodies against extracellular cell surface molecules or TCR-like antibodies [5,9,10,11]. The neoepitopes are also important for new vaccine development [12,13,14,15]. In addition, intracellular neoantigens are important targets for intrabodies. After recombinant expression of the neoantigen, purification, biochemical characterization and analysis of its function, intrabodies can be selected by phage display [16,17,18,19] (Figure 1).

To select an appropriate neoepitope for adoptive T-cell therapy and cancer vaccines, several additional evaluation steps using bioinformatics and immunological screening assays are necessary [3,20,21,22].

Intrabodies can now be selected against virtually any protein inside the cell and they have the potential to specifically inhibit the function of TAAs and even neoantigens in cancer patients. Two different kinds of intrabodies exist with different modes of action. One group of intrabodies comprises the ER intrabodies produced as scFvs inside the ER to inhibit the function of transitory proteins passing the secretory pathway [23]. Functional inhibition is performed through intrabody/antigen retention by the SE (KDEL) sequence fused to the C terminal end of the intrabody. Many ER intrabodies have been selected against overexpressed TAAs on the tumor cell surface [7,8,23].

The other group are single domain antibodies (sdAbs) comprising only the variable domain of the heavy chain VHH from camels (nanobodies) or sharks or human VH and VLs and are stable in the cytoplasm or nucleus [19]. They can be selected by phage display or ribosomal display from immune, naïve or synthetic single domain antibody repertoires [16,17,18,19] and inactivate their targets by altering their conformation or interfere with the binding of the target protein to its corresponding binding partner. sdAbs were isolated against intracellular TAAs [19], including the recent examples of hypoxia-inducible factor-1 (HIF-1) [24], serine/threonine protein kinase AKT2 [25], p53 C-terminal region involved in the interaction with Twist1 [26] and chemokine receptor US28 [27]. In addition to targeting intracellular TAAs, intrabodies have been selected against intracellular neoantigens, for example against HRASG12V [28,29,30] HRASG12V, KRASG12D, KRASG13D, NRASQ61R, KRASG12V, KRASQ61H [31] *H*- and *K*- Ras G12V [32], p21Ras [33] and KRASG12V [34].

Anti-cancer intrabodies demonstrated tumor growth inhibition in appropriate xenograft tumor mouse models [29,31,32,33,35,36,37,38,39,40,41]. Furthermore, a scFv-Fc intrabody inhibited the function of the serin (727)-phosphorylated form of STAT3 (pSSTAT3) in vitro and in mice [42]. STAT3 is involved in proliferation and apoptosis processes. CAR T cells transduced with an anti-CD7 intrabody applied in T-cell acute lymphoblastic leukemia (T-ALL) have been described [43]. The resulting CAR T cells only eliminated CD7^+^ lymphoblastic leukemia T cells and not the CAR T cells also expressing CD7.

**Figure 2:**
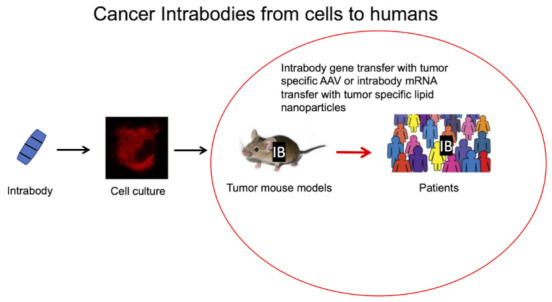
Bench-to-bedside development of anti-cancer intrabodies with the initial demonstration of tumor growth inhibition in appropriate in vitro and in vivo xenograft tumor mouse models.

However, until now, no intrabody has been applied in the clinic (Figure 2), which may be due to a lack of robust delivery methods for intrabody genes in the past. Adeno-associated viral (AAV) vectors carrying the coding region of the intrabody in combination with nanoparticles loaded with engineered mRNA have now emerged as a particularly promising strategy for intrabody targeting into tumor cells. AAV vectors are the leading gene delivery platform for gene therapy with three gene therapy drugs approved by the European Medicines Agency and the United States Food and Drug Administration [44,45,46,47].

The usage of engineered mRNA as prophylactic vaccines, therapeutic vaccines and therapeutics [48] represents a major breakthrough in efficient therapeutic drug delivery. The embedding of the mRNA in lipoprotein nanoparticles is very promising [49]. Engineered mRNA-based gene delivery avoids the risk of gene insertion into the host genome and mRNAs can be rapidly produced with modified nucleosides preventing immune activation and increasing stability and translation of mRNA [50,51]. Recently, efficient COVID-19 mRNA-based vaccines have been successfully developed using this technology [52]. To specifically deliver the intrabody to the tumor cells, which is crucial to avoid off-target effects with normal cells, AAV vectors and nanoparticles can be coated with antibodies, particularly antibody fragments, recognizing TAAs or neoantigens expressed on the cell surface of tumor cells [45,53]. Delivery of intrabodies using AAV vectors and mRNA/lipoprotein complexes may soon translate these promising molecules to the clinic.

## 2. Tumor-Associated Antigens

Two classes of tumor antigens exist: Tumor-associated antigens (TAAs)and neoantigens [1,2,54]. TAAs are expressed intracellularly or on the surface of tumor cells, can be secreted and are almost always expressed on normal tissues at lower levels relative to the tumor. Most of the antibodies and antibody fragments applied in the clinic target cell-surface-expressed TAAs [55,56]. The cell-surface-expressed antigens include receptors such as epidermal growth factor receptor (EGFR), carcinoembryonic antigen (CEA), vascular endothelial growth factor receptor-2 (VEGFR-2), insulin-like growth factor 1 receptor (IGF-1R), human epidermal growth factor receptor-2 (HER2/neu), αVβ3 integrin, mucin 5AC (MUC5AC), death receptor 5 (DR5) and programmed death-ligand 1 (PD-L1) and differentiation antigens such as *tyrosinase-related protein-2* (TRP2) and glycoprotein gp100.

The secreted antigens include the oncofetal antigen α-fetoprotein (AFP) and prostate-specific antigen (PSA). Intracellular TAAs are for example human telomerase reverse transcriptase (hTERT), RAGE-1, p53 and tissue polypeptide-specific antigen (TPS), a specific fragment of keratin 18 [1,57]. Other intracellular TAAs against which single domain antibodies already exist are capping actin protein, gelsolin-like (CapG) and BCL2-associated X (BAX), protein kinase Cɛ, c-myc, LIM domain only 2 (LMO2), endothelial and epithelial kinase (Etk), caspase-3, heterogenous nuclear ribonucleoprotein *K* (hnRNP-*K*), L-plastin, fascin, cortactin and gelsolin [19].

Furthermore tumor-associated carbohydrate antigens (TACAs) comprise glycoproteins and glycolipids which are uniquely or excessively expressed on the cell surface of tumor cells during cancer development [58]. They are difficult to target by antibodies because they often demonstrate very low immunogenicity.

## 3. Intrabodies against Oncogenic Cell Surface Receptors

TAAs passing the secretory pathway are mainly cell surface receptors. They are targeted by ER intrabodies. TAAs and neoantigens expressed in the cytosol or nucleus are targeted by single domain antibodies. ER intrabodies in the scFv format are very stable in the ER but not in the nucleus or cytoplasm whereas sdAbs are stable in both compartments [59]. ER intrabodies can be constructed from hybridoma clones as scFv or selected from human antibody phage display repertoires [8,60].

To construct intrabodies from hybridomas targeting cell surface receptors, the variable genes of the light and heavy chain of the monoclonal antibody are amplified with consensus primer, by adapter-ligated PCR, 5′ RACE or by PCR with primers of the constant region using circularized cDNA [60]. Both variable genes are then assembled to a scFv fragment and cloned into an ER targeting vector, providing the ER retention signal fused to the start of the intrabody coding region after cloning. Most anti-cancer ER intrabodies targeting cell surface receptors (human Il-2 receptor, ErbB-2, TLR2) were constructed from hybridoma clones [61,62].

ER intrabodies were also generated using phage display antibody repertoires. A scFv intrabody targeting VEGFR2/KDR was selected from an immune phage display library of mice [63]. Recently, intrabodies targeting human papillomavirus 16 E6 and E7 oncoproteins for the treatment of established HPV-associated tumors were generated from a synthetic human antibody phage display library [64]. One scFv against 16 E7 was targeted to the ER and interferes with the binding of E7 to retinoblastoma tumor suppressor (pRb). The second intrabody fused with a nuclear localization sequence rescued p53 activity, leading to cell death.

Some ER intrabodies were not targeted to cell surface receptors but to membrane proteins in intracellular compartment. These include TLR9, which translocates into the endosome after activation with CpG DNA and is involved in pancreatic tumor development [65] and polysialyltransferases ST8SiaII and ST8SiaIV localized in the Golgi apparatus which are activated during the growth of several tumors [66]. All those intrabodies were generated from hybridoma clones [35,67].

## 4. Intrabodies against Cytoplasmic or Nucleus Located TAAS

Several intrabodies have been generated in recent years against intracellular antigens [19], (see Section 3). The sdAb against F-actin capping protein CapG inhibited breast tumor metastasis in a xenograft tumor mouse model [36]. Recently an anti-HIF-1α nanobody was developed to decrease gemcitabine resistance in pancreatic cancer patients [24]. The intrabody competitively inhibited the binding of the transcription factor HIF-1α heterodimer to the aryl hydrocarbon receptor nuclear translocator (ARNT), leading to the inhibition of the HIF-1/VEGF pathway in vitro. It is known that high HIF-1α expression reduces sensitivity to gemcitabine, which is applied to treat pancreatic adenocarcinoma and other cancers [68]. Interestingly the nanobody reduced tumor growth in mice treated with the nanobody to 41.58%, treated with gemcitabine to 64.89% and treatment with both gemcitabine and nanobody to 80.44%, respectively.

In another approach, nanobodies against AKT2, an isoform of the three AKT isoforms (AKT1, AKT2 and AKT3) of serine/threonine protein kinase AKT (also known as protein kinase B), were developed. AKT regulates cell proliferation, survival, growth, migration and metabolism and is hyperactivated by phosphorylation in cancer [69]. One nanobody (Nb8) affects the phosphorylation and/or expression levels of a wide range of proteins downstream of AKT, resulting in a G0/G1 cell cycle arrest, the induction of autophagy, a reduction in focal adhesion count and loss of stress fibers.

Furthermore, a nanobody was recently reported to inhibit the interaction between Twist1 and p53 [26]. Twist1 facilitates the degradation of p53 by the negative regulator murine double minute 2-Protein (MDM2), which in wild-type cancer inhibits the transcriptional activity of p53 and induces its degradation. This study demonstrated that targeting Twist1/p53 interaction with nanobodies might be a therapeutic option.

Interestingly, the function of a new nanobody against a G protein-coupled receptor (GPCR) of the human cytomegalovirus (CMV), US28, which is involved in cancer progression in glioblastoma was demonstrated [27]. The nanobody (VUN103) inhibits constitutive US28 signaling by G protein displacement and inhibited US28-enhanced spheroid growth in glioblastoma tumor cells. Phosphorylation of STAT3 was completely inhibited to the level of uninfected cells in glioblastoma tumor cells infected with CMV and transduced with the VUN103 intrabody. The group developed another nanobody (Nb7) which binds ligand-bound US28 and inhibits signaling too. Both nanobodies recognize two different conformations of US28, the super activated US28 induced by ligand CX3CL1 and the constitutively active US28. In both cases, the binding of the nanobodies inhibited the recruitment of the G protein αq subunit (Gαq).

Furthermore, a sdAb ER intrabody targeted an ER membrane receptor: the human immunoglobulin heavy chain (VH) targeted B-cell receptor-associated protein 31 (BAP31 and blocked cyclin kinase inhibitor p27kip1 degradation, leading to growth inhibition and apoptotic death in GC cells and GC tumors in mice [40]. Finally, a novel nucleus-located intracellular nanobody against HPV16 E6 oncoprotein was recently generated from an immune camelid single domain library, inhibiting tumor growth in HPV16 mouse models [40].

## 5. Neoantigens

Neoantigens arise from somatic mutations and are specific for tumor cells. Most neoantigens reside in the cytoplasm or nucleus of the cell where they enhance tumor cell growth [70]. Peptides of these cancer-specific aberrant proteins are presented in complex with MHCI or MHCII on the cancer cell surface and can be recognized by appropriate CD8^+^ T cells and CD4^+^ T cells, inducing an immune response and tumor cell killing [71]. The neoantigens can arise from single nucleotide variants (SNV). Frameshift insertion–deletion (indels) mutations result in a novel amino acid sequence downstream of the indel. In addition, new sequences can be generated after chromosomal translocations or derived from splice variants [2,54]. Two groups of neoantigens have been found. Public or shared neoantigens are expressed in more than one patient and private antigens are only observed in a single patient. Shared neoantigens arise from frequent fusions and mutations within hotspot regions and are found in important driver oncogenes. Examples are the RUNX1-RUNX1T1 fusion [72] and exon 12 mutations in nucleophosmin1(NPM1) in acute myelogenous leukemia [AML] [73]. On the contrary, “private neoantigens” comprise patient-specific mutations and fusions and are localized more frequently in loci non-essential for tumorigenesis and metastasis, termed “passenger” mutations. An example is recently found mutations in several genes from patients with small cell lung cancer (NSCLC) [74].

Some mutations and fusions in neoantigens are specific for one particular cancer type. Other mutations and fusions occur in oncogenes or tumor suppressor genes of numerous cancers. Public neoantigens of TP53 [75] and the members of the Ras-MAPK pathway [76] are found in several cancers. The most recurrent oncogenic mutations of the RAS genes (NRAS, KRAS, HRAS) across different cancers have been found at codons 12, 13 and 61. The neoantigen peptides derived from the mutated genes of TP53 and RAS are very attractive epitopes as targets for adoptive T-cell therapy and the proteins are also attractive targets for intrabodies.

### Identification of Neoantigens for Adoptive T-Cell Therapy, Cancer Vaccines and Intrabodies

Neoantigen identification is routinely performed by several sequential steps: Neoantigens are identified by whole-exon sequencing with high efficiency, wide coverage and low false negative rate [77,78]. Alignment of exome DNA and RNA sequencing (RNA-seq data of tumor cells and normal cells, somatic and germline variant detection and RNA-seq expression estimation leads to expressed neoantigen transcripts [20]. The identified characterized neoantigens can be directly used for the selection of appropriate intrabodies using phage display antibody repertoires (Figure 1).

For the usage of neopeptides as cancer vaccines and for adoptive T-cell therapy, candidate peptides will be analyzed for the binding of the mutant peptide regions to the patients MHC alleles. MHC binding affinity, clonality and distinction from self-antigen can be determined by bioinformatics [79,80]. In parallel, MHC/peptide complexes can be purified from tumor tissue, effector T cells identified and activation of T cells with antigen presenting cells and tumor cells expressing the appropriate MHC/peptide complexes assessed [3,20]. To identify neoepitope-specific effector T cells, antigen-presenting cells can be transduced with a candidate peptide library or viral minigene library of the selected peptide candidates and then co-cultured with tumor-infiltrating T cells [81]. Finally, identified T cells will be evaluated in regard to the recognition of autologous tumor cells.

## 6. Intrabodies against Intracellular Neoantigens

Intracellular TAAs and neoantigens are targeted by single domain antibodies selected from camels, sharks or human VH or VL antibody repertoires by phage display or ribosomal display [16,17,19]. Recently a new technique was established for the selection of small molecules, peptide aptamers and cytoplasmic intrabodies: localization-based interaction screening (SOLIS) [82]. SOLIS employs two chimeric proteins in which a membrane localization motif (CaaX) is fused at the C-terminus of interest neoantigens while the catalytic domain of SOS is fused at the C-terminus of another protein of interest. Son of sevenless (SOS) is a guanine nucleotide exchange factor that activates Ras upon growth factor stimulation. Interaction between the two proteins of interest induces membrane localization of the SOS fusion protein and then cell proliferation due to the activation of the RasMAPK signaling pathway.

**Figure 3:**
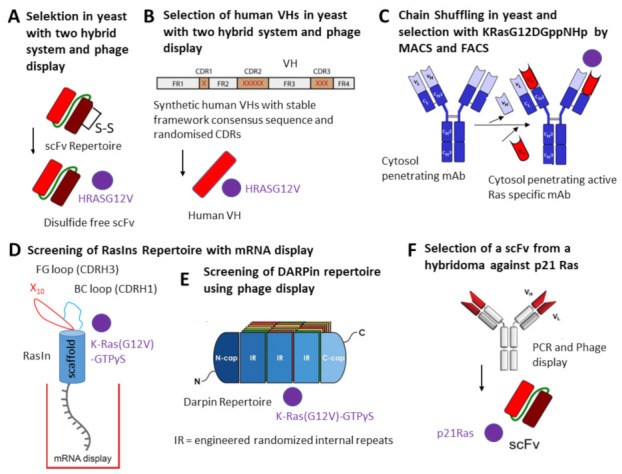
Strategies used to select intrabodies against Ras mutants.

Intrabodies were selected against oncogenic Ras mutants using different approaches leading to several intrabodies in different formats: disulfide free scFv, human VH nanobody, complete IgG antibody, “RasIns” (fibronectins) and DARPins [28,29,30,31,32,33,34] (Table 1) (Figure 3). In its active form, Ras binds to GTP and can interact with multiple effector proteins such as Raf kinases, PI3K (phosphatidylinositol 3-kinase) and RalGDS (Ral guanine nucleotide dissociation stimulator) [83]. Ras mutants of the canonical *ras* gene family, *H-ras*, *N-ras* and *K-ras,* are frequently activated by point mutation in human cancers, predominantly at G12, G13 and Q61 residues, leading to impaired GTPase activity resulting in constitutively active mutants persistently binding GTP and promoting tumorigenesis and tumor malignancy [84]. KRAS G12V is present in 3.47% of AACR GENIE cases, with pancreatic adenocarcinoma, lung adenocarcinoma, colon adenocarcinoma, colorectal adenocarcinoma and rectal adenocarcinoma having the greatest prevalence. The proteins *H-ras*, *N-ras* and *K-ras are also called H*-p21Ras, *K*-p21Ras and *N*-p21Ras.

Tanaka and Williams selected scFvs recognizing oncogenic HRASG12V using the intracellular antibody capture (IAC) technology [28]. A stable disulfide-free scFv was selected and its expression optimized by exchanging the VH and VL framework with a known stable consensus framework yielding high expression in bacteria and mammalian cell cytoplasm. When NIH 3T3 cells were co-transfected with the disulfide-free scFv and a RAS mutant-dependent luciferase construct the number of transformed foci was reduced to 30%. In a following approach, a human VH domain was selected from two synthetic VH domain libraries with fully randomized complementarity determining regions (CDRs) introduced into a single stable intrabody framework [28,29]. One human VH was selected in yeast. This VH binds to activated GTP-bound wild-type HRAS and HRAS (G12V). It was shown that the sdAb inhibited tumor growth and metastasis in a tumor xenograft mouse model. Nude mice were injected subcutaneously with mouse (NIH3T3-EJ) or human (HT-1080 or DLD-1) tumor cells transduced with the anti-Ras intrabody. Tumors were not developed in mice when the sdAb was expressed in the tumor cells compared to cells with an empty vector or an irrelevant intrabody. The crystal structure of intrabody and Ras showed that the intrabody covers the surface of RAS where most of the effectors (RAF, RALGDS and PI3K) interact.

Another approach utilizes antibody-like ligands exhibiting an immunoglobulin-like fold that are composed of the 10th fibronectin type III domain of human fibronectin, with two hypervariable loops that are structurally similar to antibody CDRH1 (BC loop) and CDRH3 (FG loop) regions. The RasIn1 and RasIn2 antibody-like ligands were developed as intrabodies that recognize active, GTP-bound *K*- and *H*-Ras and the corresponding G12V mutants [30]. The antibody-like ligands were selected by mRNA display using as antigen *K*-Ras (G12V)-GTPγS and a library comprising a modified 10FnIII scaffold containing a randomized biased CDR1 sequence derived from iDab#6 [29] and a naive randomized sequence of CDR3. Detailed binding analysis demonstrated that RasIn1 and RasIn2 recognized the binding domain of the Raf kinase in activated *H*-RasG12V

A very interesting study resulted in the human IgG intrabody RT11 against Ras mutants [31]. This anti-Ras mutant antibody was engineered from a human antibody previously generated and is internalized through clathrin-mediated endocytosis using heparan sulfate proteoglycan (HSPG) as a receptor and escapes from early endosomes into the cytosol [84]. The intrabody recognizes the GTP-bound active forms of wild-type (WT) KRas, NRas and HRas and their oncogenic mutants with mutations at positions 12, 13 or 61, such as KRasG12D, KRasG12V, KRasG13D, KRasQ61H, HRasG12V and NRasQ61R. Inactive GDP bound Ras WT and mutants were not recognized. To target the intrabody to tumor-associated integrins, the RGD10 cyclic peptide (targeting α_v_-integrins) was genetically fused to the light chain of the intrabody (RT11i). It was shown that RT11-i significantly inhibits the tumor growth of oncogenic Ras mutant tumor xenografts in mice.

The work was continued by the same group by engineering a new human IgG intrabody (inRas37) binding to activated GTP-bound Ras mutants with two-fold stronger activity [32]. The endosomal escape was higher compared to the previously published intrabody RT11i and the cytosolic concentrations were two-fold higher in targeted cells. The inhibition of tumor growth was seen in several xenograft tumor mice bearing different preestablished colorectal tumors. Combination of an inhibitor of Yes-associated protein 1 (YAP1, a transcription factor which regulates cancer cell proliferation) and inRas37 showed a synergistic effect inRas37-sensitive tumor cell lines.

An anti-p21 Ras scFv antibody was generated from a hybridoma recognizing wild-type *H*-p21Ras, *K*-p21Ras and *N*-p21Ras [33] and their mutated variants. The intrabody significantly inhibited the tumor growth of nude mice with established tumors derived from a human colon cancer cell line SW480 or a human liver cancer cell line BEL-7402. Intrabody was directly injected into the tumor by adenoviral gene transfer. Recently it was shown that the genetic fusion of the RGD4C peptide binding the integrin αvβ3 to the C-terminus of the anti-p21 Ras scFv, which could not penetrate the cell membrane alone, resulted in the penetration and growth inhibition of the human colon cancer cell line SW480 in vitro [87].

Recently, two other antibody-like ligands of the ankyrin repeat protein type (DARPins) [88] were selected that bind to an allosteric site of GDP- or GTP-bound KRASWT and KRASG12V, inhibiting KRAS nucleotide exchange and dimerization [34]. DARPins were isolated from a phage display library by biopanning using biotinylated KRASG12V.

Non-small cell lung cancer (NSCLC) is heterogeneous and KRAS G12C is the most prevalent of the KRAS mutations. Therapies targeting KRAS are beginning to show clinical potential, most notably with KRASG12C inhibitors [89,90]. So far, other gene variants have not been targeted in advanced clinical studies. Therefore, using intrabodies that have been selected against intracellular neoantigens, for example against HRASG12V [28,29,30] KRASG12D, KRASG13D, KRASQ61H, KRASG12V, NRASQ61R [31], *H*- and *K*-Ras G12V [32], p21Ras [33] and KRASG12V [34], might be very important for developing advanced phase clinical studies.

Nevertheless, extensive intratumor heterogeneity (ITH) related to genetic diversity both within individual tumors and between primary and metastatic tumors for different cancer types has been newly discovered [91]. Peptide vaccines may only kill a small number of tumor cells if the neoantigens targeted are derived from mutated subclones. Similarly, intrabodies may also be ineffective if new driver neoantigens are elicited during treatment. In the future, it is essential to identify effective and common neoantigens containing tumor driver mutations. It might be possible to apply a mixture of intrabodies targeting different driver neoantigens or combine the intrabody with a small molecule inhibitor against another oncogenic neoantigen identified inside the tumor.

Mutation-derived neoantigens can be recognized by T cells and increased mutated neoantigens (neoantigen burden) influence the survival across diverse types of human cancers. Tumors with both a high clonal neoantigen burden and low neoantigen intratumoral heterogenicity encourage longer survival. Furthermore, a relationship between neoantigen burden and response to immune checkpoint inhibitors has been demonstrated too. Sensitivity to PD-1 and CTLA-4 blockade in patients with advanced NSCLC and melanoma was enhanced in tumors enriched for clonal neoantigens [92,93,94]. In this context, the treatment of anti-neoantigen intrabodies with immune checkpoint inhibitors may increase therapeutic efficiency.

## 7. Bringing Intrabodies into Cancer Patients: Delivery of Intrabodies with Nanoparticles or AAV

**Figure 4:**
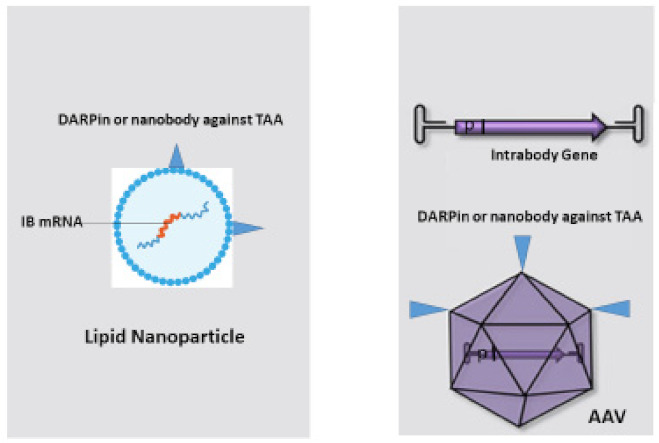
Tumor-cell-specific intrabody transfer with nanoparticle or AAV, *p* = promoter.

Many methods for intrabody delivery into target cells are available such as the delivery of intrabody genes by plasmids or viral transduction, or using polymeric and dendrimeric nanoparticles embedded with small hydrophobic and hydrophilic drugs, peptides, vaccines and antibodies [95]. The most promising and efficient methods for intrabody transduction and expression in cancer cells are the use of lipid nanoparticles with embedded mRNA [49,96,97,98] and transduction with a new generation of AAVs carrying the intrabody cDNA [45,47] (Figure 4).

### 7.1. Nanoparticles Embedded with Intrabody mRNA

mRNA can be used as prophylactic vaccines, therapeutic vaccines or therapeutics [48]. Recently, the proof of principle of the mRNA technique was very successfully given by COVID-19 vaccines and VEGF mRNA for the regenerative treatment of heart failure [52,99]. Therapeutic vaccine mRNA is currently focused on immuno-oncology. Two examples are the mRNA-5671 developed by Moderna/Merck and partners for cancers involving KRAS Mutations and the drug BNT113 developed by BioNTech for HPV-16-caused cancers. More mRNA therapeutics are in the pipeline, for example it was shown that a mRNA-encoded bispecific CD3 x CLDN6 antibody eliminated human ovarian carcinoma xenografts in NSG mice engrafted with human PBMCs [100]. Recently a phase 1 trial with a mRNA encoding a CHIKV neutralizing antibody (CHKV-24 IgG), administered by intravenous infusion in adults, generated dose-dependent increases in CHKV-24 IgG at levels which should provide protection against human infection and disease [101].

Modifications of the mRNA to improve mRNA translation efficiency and to reduce immunogenicity has established the mRNA technology as suitable for usage as therapeutics. mRNA can be modified with the alternative nucleotides pseudouridine (ψ), 1-uridine and cytidine, which eliminates the activation of intracellular signaling by PKR and RIG-I and leads to enhanced protein expression [102]. Other factors that can be optimized are the cap structure [103] and poly(A) lengths [104], leading to a higher amount of protein production. UTRs [105] and codon optimization [106] can also improve the mRNA translation or half-life. Furthermore, the removal of aberrant RNAs generated by in vitro transcription by high-performance liquid chromatography (HPLC) can reduce the activation of innate immunity [107].

Transduction of mRNA with nanoparticles has been optimized in recent years. mRNA possesses a highly negative charge. The negative charge can be shielded by a positive charge to bring the mRNA across the membrane with its negative potential. In addition, the mRNA must be protected against degradation by nucleases. Therefore, the delivery of mRNA is commonly performed with nanoparticles composed of pH-responsive lipids or cationic lipids bearing tertiary or quaternary amines to encapsulate the polyanionic mRNA [49]. In addition, neutral helper lipids to stabilize the lipid bilayer of the lipid nanoparticles and a polyethylene glycol (PEG)-lipid are incorporated to improve the colloidal stability and forming a hydration layer over the nanoparticles. Solid liquid nanoparticles (SLNs) comprising solid lipids and nanostructured lipid carriers (NLCs) comprising mixtures of solid and liquid-crystalline lipids have been developed as alternatives to common liposomes, which comprise liquid-crystalline lipid bilayers [97]. They demonstrate enhanced physical stabilities, high loading capacities and facile production on a large scale and have been used for the delivery of several therapeutics [108].

To specifically target antibody/intrabody mRNA to tumor cells, nanoparticles can be conjugated with anti-TAA antibodies [109]. Particularly polymeric nanoparticles have been conjugated with different antibodies for imaging and therapy [110] and antibody-conjugated liposomes have been developed to improve the blood residence time and targeted delivery efficiency [111]. The antibody formats fused to the nanoparticles are complete monoclonal antibodies and mostly recombinant antibody fragments such as Fabs, scFvs, bifunctional and bispecific antibodies and nanobodies [53]. Concerning recombinant antibody fragments, very promising results have been obtained with nanobodies due to their small size and stability. EGFR and HER-2 nanobodies demonstrated very efficient targeting in cancer therapy. Meel et al. delivered an anti-(IGF)-1R kinase inhibitor AG538 by anti-EGFR nanobody-liposomes to cancer cells and this targeting resulted in strong antiproliferative activity [112]. Biocompatible and biodegradable polymersomes were functionalized with an anti-Her2 nanobody and the functionalized nanoparticles were able to specifically target breast cancer cells expressing HER2 receptors [113]. Furthermore, an approach of cell-specific mRNA transfer with polymeric nanocarriers was shown by polymeric mRNA nanocarriers fused with anti-CD3 antibody fragments that were simply mixed with CAR T cells to reprogram them via transient expression [114].

### 7.2. Delivery of Intrabody Genes by AAV

Recombinant AAV vectors are the most promising vectors for gene therapy. They are able to deliver gene-editing enzymes, RNA interference and antibodies [47]. AAV capsids have been generated with cell/tissue specificity, which demonstrated improved transduction efficiency and reduced immunogenicity. These new AAV vectors are under evaluation in pre-clinical and clinical trials [115,116]. New AAV vectors have been selected by direct evolution or rational design.

#### 7.2.1. Direct Evolution

A detailed study of all single codon mutants of the AAV2 cap gene concerning virus production and in vivo delivery in mice was recently published [117]. The cap gene comprises the overlapping genes of VP1, VP2 and VP3 which build up the capsid of AAV. Following direct evolution, AAV libraries with targeting random peptide insertions into exposed surface loops of different AAV serotypes has been performed [118,119,120]. Different peptides can be inserted into exposed surface loops of AAV2 without the alteration of the capsid assembly and new AAV2 mutants have been generated which can be retargeted to alternative cell types and which avoid antibody neutralization [118,121,122]. At the moment, random peptide insertions of 7–12 amino acids at AAV2 cap position 587/588 are widely used. Recently, 23 pre-selected peptide sequences comprising 7–9 amino acids were inserted in a designated capsid surface loop variable region VIII (VR-VIII, amino acids 580–595, also comprising cap 587/588 position, see above for 13 AAV serotypes) [118]. The major finding was that highly efficient capsids could be identified by transduction with many different cell lines. It was clearly demonstrated that AAV serotypes, other than AAV2, can be used as scaffolds for peptide display to select new very-transduction-efficient AAV vectors. The cap genes of the AAV variants were fused to YFP and the transduction rate was easily analyzed by the determination of the percentage of virus-transduced YFP-expressing cells. Interestingly, new AAV2 variants were selected by in vivo biopanning of random virus display peptide libraries [123]. They showed that vectors displaying peptides obtained after in vivo selection have a significantly improved the transduction profile in breast cancer or lung tissues after systemic administration.

#### 7.2.2. Rational Design

Rational design involves the direct modification of an existing AAV serotype capsid by cell surface receptor binding molecules genetically or via covalent or non-covalent coupling called transductional targeting. Transcriptional targeting allows the expression of the transgene primary in cells comprising the appropriate tissue-specific promoter due to the incorporation of an appropriate tissue-specific promoter into the AAV genome [45]. Furthermore, in post-transcriptional targeting, microRNA targeting sites are incorporated into the AAV genome, which results in the degradation of the microRNA sequences containing mRNA in cells producing the corresponding microRNA [124]. Generally, in tumor cells, the production of microRNA is downregulated compared to normal cells.

##### Transcriptional Targeting

For transcriptional targeting, promoters are applied that are predominantly active in cancer cells but silent in normal cells. For example, survivin is highly expressed in breast and lung cancer and has antiapoptotic effects and promotes tumor angiogenesis [125]. Another example is the telomerase reverse transcriptase (TERT), which is aberrantly expressed via its promoter in approximately 90% of aggressive cancers and 73% of tumor cases and is able to upregulate telomerase activity in cancer cells leading to tumor growth [126].

Tumor angiogenesis leads to nonfunctional blood vessels, the reduction in blood supply and low oxygen concentration in the tumor microenvironment (hypoxia) [127]. Hypoxia-inducible factors (HIFs) can increase the proliferation of tumor cells by the regulation of expression of corresponding genes. Therefore, hypoxia is a target for tissue-specific gene therapy in cancer. To guarantee tumor-specific transgene expression, hypoxia-response elements (HREs) that serve as the binding site of HIFs localized in the promoter/enhancer regions of hypoxia-induced genes (for example: Epo, VEGF-A, PGK1) have been combined with tumor-specific promoters. An example is the hypoxia/radiation dual sensitive chimeric HRE/early growth response 1 (Egr 1) promoter. This construct induces the expression of the proapoptotic second mitochondria-derived activator of caspases (Smac) gene and enhances radiation-induced A549 human lung adenocarcinoma cell death under hypoxia [128].

Bioinformatics was used to select appropriate cancer-cell-specific transcription factors and for the design of specific mini-promoters to improve cancer gene therapy [129]. Tumor-specific mini-promoters comprise a combination of tumor-specific transcription factor response elements (TFREs) to enhance gene expression levels in tumor cells and reduce side effects [129]. Ho et al. constructed a synthetic mini-promoter comprising binding sequences of HIF-1α, cAMP response element-binding protein (CREB) and NF-κB. This D5 promoter led to the overexpression of the reporter gene hrGFP in tumor tissues but not in normal tissues. Mice-bearing B16F10 melanoma cells were intravenously injected with the therapeutic gene RBDV-IgG1 Fc that encodes for a fusion protein of VEGF-A and the Fc region of human IgG1 encapsulated into a liposome-PEG-PEI complex. RBDV-IgG1 Fc can bind to VEGF receptor 1 or 2, inhibiting tumor angiogenesis. It was demonstrated that the expression of RBDV-IgG1 Fc via the new synthetic promoter could block tumor angiogenesis.

A very interesting approach was recently developed by Dai et al. [130]. They developed a cancer-cell-specific NF-κB-activated gene expression vector. In general, tumor cells are expressing high levels of NF-κB compared to normal cells. Construction of a weak NF-κB promoter by fusing a NF-κB decoy sequence with a minimal promoter to express downstream effector genes should inhibit tumor growth without affecting normal cells. As a proof of principle, CRISPR/Cas9 was activated via the new NF-κB promoter. Co expression of a telomere-targeting sgRNA (TsgRNA) resulted in the cleavage of telomeric DNA by CRISPR/Cas9 and in vivo tumor growth inhibition. This was demonstrated in mice-bearing hepatoma Hepa 1–6 cells injected intravenously with a recombinant AAV carrying the new developed vector comprising the synthetic NF-κB promoter, CRISPR/Cas9 and telomere-targeting sgRNA (TsgRNA).

##### Transductional Targeting

Transductional targeting aims to specifically deliver AAVs to tumor cells by modulating the capsid surface. A prerequisite is to abolish binding to receptors naturally used for cell attachment and internalization. Noncovalent coupling of cell surface receptor binding molecules can be achieved with bispecific antibodies which recognize the residues of the capsid and a cell-specific receptor on cancer cells [131] or by chemical coupling of biotin to amino groups of capsid surface-exposed arginine (R) or lysine (K) residues which after modification could bind to avidin-linked receptor binding peptides [132]. With reference to bispecific antibodies, a new bispecific antibody-based platform for retargeting of capsid-modified AAV vectors was recently established [131]. A short peptide epitope derived from an alpha helical region of proprotein-convertase subtilisin/kexin type 9 (PCSK9, 2E3 epitope) recognized by a monoclonal antibody (2E3) was inserted into different regions of the AAV2 surface. Insertion of the epitope abolished the binding to heparin sulfate–proteoglycan (HSPG). Incubation of AAV vectors with bispecific antibodies binding to 2E3 and fibroblast activation protein (FAP) or to programmed death-ligand 1 (PD-L1), respectively, retargeted the new AAV2 capsid to FAP, which is upregulated on activated fibroblasts within the tumor stroma and to PD-L1, which is strongly upregulated on the surface of tumor cells in many cancers.

Genetic capsid modification for specifically retargeting AAV to tumor cells by the incorporation of DARPins, nanobodies and receptor-specific peptides has been demonstrated. DARPins and nanobodies [45,46,133] are the most suitable antibody formats because they are correctly folded during the assembly of the AAV capsid in the nucleus, in contrast to scFvs. DARPins against CD4, Her2/neu and EpCAM were incorporated into the capsid of AAV to specifically transduce AAV into mice [46]. Therefore, myc and his-tagged DARPins were fused to the *N*-terminus of VP2 and arginines R585 and R588 were mutated to alanine to prevent binding to heparin sulphate proteoglycan. The treatment of mice injected with luciferase-labelled breast-cancer-derived MDA-MB-453 cells into the tail vein with IMAC-purified HER-AAV delivering the cytotoxic gene herpes simplex virus (HSV) thymidine kinase (TK) leads to reduced tumor burden, before relapse occurred. HSV TK phosphorylates given ganciclovir (GCV), which afterwards is converted to GCV triphosphate, incorporated into DNA and stops replication. Interestingly mice treated with Her2-AAV survived significantly longer than Trastuzumab-treated mice.

In a recent published work, nanobodies were used to retarget AAV2 to CD4^+^ cells. In total, five anti-human CD4 nanobodies were inserted into the hypervariable loop of the GH2/GH3 surface of VP2 or *N*-terminus of the VP1 capsid protein [134]. Arginines R585 and R588 were mutated to alanine to prevent binding to heparin sulphate proteoglycan. Primary human CD4^+^ cells and CD4^+^ T lymphocytes from PMBCs were transduced by the nanobody-modified capsid variants with different efficiency. These newly developed nanobody-modified AAV2 variants are promising vectors in cases where the delivery of nucleases or recombinases into CD4^+^ lymphocytes is requested, for example in therapeutics approaches against HIV.

Different peptides have been inserted into the AAV capsid to target tumor cells. For example, the RGD4c peptide (CDCRGDCFC) has enabled the transduction of tumor cell lines via ανß3 or ανß5 integrin receptors [135]. These modified capsids could also target proliferating endothelial cells in tumor vessels [136].

Nevertheless, despite the promising developments of transfection/transduction with specific nanoparticles or specific AAVs, some critical points have to be mentioned. Human immune responses can be previously elicited against WT AAV to which over 90% of humans are exposed. This could limit recombinant AAV transduction and reduce long-term transgene expression [137,138]. The application of exosome-enveloped AAV could prevent AAV capsid recognition by CD8^+^ cells and Toll-like receptors and neutralization by antibodies resulting in higher transduction efficiency [139]. Exosomes are very promising tools for the delivery of RNAi, mRNA or DNA for cancer therapy [140,141].

Referring to nanoparticles, the tumor microenvironment of solid tumors could represent a barrier which could lead to inefficient penetration by nanoparticles. A meta-analysis demonstrated that, on average, only 0.7% of injected NP doses reach tumors [95]. In addition, TAA targeting can lead to off-target effects because TAAs which are overexpressed in tumor cells are also often expressed in healthy tissue. One way to prevent expression of transgenes in healthy cells is combining transcriptional and transductional targeting using AAVs. The selectivity of transgene expression has been demonstrated by adenovirus targeting ovarian tumors [142].

To improve immunotherapy with intrabodies combination of this strategy with other immunotherapy approaches are possible. For example, application of intrabodies combined with antibodies blocking immune checkpoint molecules [143] to expand CD8^+^ T-cells and T_H_1-T cells. Thereby addition of an double-stranded (ds)RNA adjuvant incorporated into poly (lactic-co-glycolic acid) (PLGA) particles cold enhance activation of T-cells [144]. Possible is also to evaluate a combination of intrabodies with TAA specific monoclonal antibodies or immunozytokines, bispecific antibodies, immunotoxins or CAR T-cells.

## 8. Conclusions

Intrabodies are very potent molecules to inhibit the function of overexpressed TAAS or mutated neoantigens on the cell surface or in the cytoplasm and nucleus. This has been demonstrated in cell culture as well as in xenograft tumor mouse models [29,31,32,33,35,36,37,38,39,40,41]. The reason why the intrabodies are very attractive for cancer immune therapy is their highly specific inhibition of TAA or neoantigen function without interfering with other intracellular molecules inside tumor cells as it might be possible by RNAi or CRISPR-Cas in in vitro experiments but not applicable in an in vivo human setting due to off-target effects [145,146,147].

The identification of cancer mutations which are uniquely expressed in tumor cells has increased enormously in the last ten years [54] and some intrabodies already exist against some neoantigens [28,29,30,31,32,33,34]. Now intrabodies against almost every TAA or neoantigen can be selected by phage display or ribosomal display using antibody repertoires comprising scFvs, nanobodies, shark antibodies, human VHs and VLs or DARPins [8,16,17,19,148,149] and evaluated in appropriate xenograft tumor mouse models. Conferring to tumor mouse models it would be very useful to analyze the effect of intrabodies in immunodeficient or humanized mice transplanted with human tumor tissue. This would enable us to study the effect of the intrabody on tumor progress in more detail, including the tumor microenvironment and tumor heterogeneity [150].

At the moment, the biggest challenge in translating TAA/neoantigen-directed intrabodies into the clinic is the specific targeting of the intrabodies to the tumor cells. The promising development of tumor-specific lipid nanoparticles which could be embedded with an mRNA transgene or new capsid-modified and tumor-specific recombinant AAVs should enable tumor-cell-specific intrabody transfection/transduction in cancer patients [45,47,49,53,96,97,98,109] and may finally bring intrabodies into the clinic.

## Figures and Tables

**Table 1:** Reports of intrabody-mediated knockdown of the *ras* gene family.

Target	Selection of Intrabody	Physiological Knockdown Readout	Ref.
HRASG12V	A disulfide free scFv was selected with the intracellular antibody capture (IAC) technology [85].	When NIH 3T3 cells were co-transfected with the disulfide-free scFv and RAS mutant-dependent luciferase construct the number of transformed foci was reduced to 30%.	[28]
HRASG12V	A human VH domain was selected from two synthetic VH domain libraries with fully randomized complementarity determining regions (CDRs) introduced into a single stable intrabody framework. One human VH was selected in yeast.	The VH binds to activated GTP-bound wild-type HRAS and HRAS (G12V). Nude mice were injected subcutaneously with mouse (NIH3T3-EJ) or human (HT-1080 or DLD-1) tumor cells transduced with the anti-Ras intrabody. Tumors were not developed in mice when the sdAb was expressed in the tumor cells compared to cells with an empty vector or an irrelevant intrabody.	[29]
GTP-bound *K*- and H-Ras and the corresponding G12V mutants	Antibody-like ligands as intrabodies were developed (RasIn1 and RasIn2). They were selected by mRNA display using an antigen *K*-Ras(G12V)-GTPγS.	Detailed binding analysis demonstrated that RasIn1 and RasIn2 recognized the binding domain of the Raf kinase in activated *H*-RasG12V.	[30]
Ras mutants	This anti-Ras mutant antibody was engineered from a human antibody previously generated and is internalized through clathrin-mediated endocytosis using heparan sulfate proteoglycan (HSPG) as a receptor and escapes from early endosomes into the cytosol [86].	The intrabody (RT11-i) recognizes the GTP-bound active forms of wild-type (WT) KRas, NRas and HRas and their oncogenic mutants with mutations at positions 12, 13 or 61, such as KRasG12D, KRasG12V, KRasG13D, KRasQ61H, HRasG12V and NRasQ61R. RT11-i significantly inhibits the tumor growth of oncogenic Ras mutant tumor xenografts in mice.	[31]
Ras mutants	A new human IgG intrabody (inRas37) binding to activated GTP-bound Ras mutants with two-fold stronger activity was engineered from RT11-i.	Inhibition of tumor growth was seen in several xenograft tumor mice bearing different preestablished colorectal tumors.	[32]
p21 Ras	scFv antibody was generated from a hybridoma.	The scFv recognizes wild-type *H*-p21Ras, *K*-p21Ras and *N*-p21Ras [32] and their mutated variants. The intrabody significantly inhibited the tumor growth of nude mice with established tumors derived from human colon cancer cell line SW480 or human liver cancer cell line BEL-7402.	[33]
KRASG12V	DARPins were selected from a phage display library by biopanning using biotinylated KRASG12V.	DARPins bound to an allosteric site of GDP or GTP-bound KRASWT and KRASG12V inhibiting KRAS nucleotide exchange and dimerization.	[34]

## Data Availability

No new data were created or analyzed in this study. Data sharing is not applicable to this article.
